# Enhancing reading abilities of learners with intellectual impairments through computer technology

**DOI:** 10.4102/ajod.v6i0.206

**Published:** 2017-07-24

**Authors:** Cina P. Mosito, Albert M. Warnick, Emmanuel E. Esambe

**Affiliations:** 1 Faculty of Education, Cape Peninsula University of Technology, South Africa; 2 Molenbeek School for LSEN, Maitland, South Africa; 3 Academic Literacy, Fundani, Cape Peninsula University of Technology, South Africa

## Abstract

**Background:**

Developments in the teaching of children with disabilities support pedagogy that emphasises learners’ strengths as opposed to their assumed deficiencies. Educators and mediators who advocate this view continually strive for tools and methodologies that enhance learner participation in academic environments. Computer technology is one of the tools recognised for its potential to enrich learning experiences of learners with an intellectual impairment.

**Objectives:**

We sought to assess the influence of text-to-speech stories on the reading ability of intellectually challenged learners.

**Method:**

A qualitative action research study that involves learners at a special school in Cape Town, South Africa. Pre- and post-test data of the reading performance of learners are analysed with a focus on how they demonstrate change.

**Results:**

Although no claims can be made about the explicit influence on reading performance, computer-assisted learning has the potential in isolating reading processes that classroom-based interventions can address. In addition, computers enhance motivation and enthusiasm to learn.

**Conclusion:**

A need for education based on inclusion and positive differentiation remains the key driver in any educational interventions.

## Introduction

A diagnosis of intellectual disability (ID) invites several questions on the extent to which one can learn and the speed at which learning can take place (Baroff & Olley 2012:1). ID has been noted to entail impairments that significantly affect (1) conceptual (language, reading, writing, mathematics, reasoning, knowledge and memory) (Alfassi, Weiss & Lifshitz 2009; Edyburn 2004), (2) social (empathy, social judgment, interpersonal communication skills, the ability to make and retain friendships, and similar capacities) and (3) practical (self-management in areas such as personal care, job responsibilities, money management, recreation, and organising school and work tasks) domains (American Psychiatric Association 2013a; Salvador-Carulla et al. 2011). The abilities affected by the ID suggest that knowledge and skills acquisition for learners with disability are often characterised by a higher level of challenges when compared to learners without the condition (Lesgold & Welch-Ross 2012). Given the wide-ranging challenges of this group of learners, the following question is worth pursuing:

What kind of change in reading abilities do learners with intellectual disability undergo when taught through text-to-speech books?

Technological resources can be used to support the learning of the intellectually disabled learner (Phillips et al. 2008). Technology, in particular the physical properties of colour, sound and imagery of computers can enhance a learner’s understanding of language within a particular context (Chai, Vail & Ayres 2014). In fact, some researchers have pointed out that learners who are placed in conditions of computer-assisted teaching of literacy concepts outperform learners who are under the guidance of educators alone (Campbell & Mechling 2009) and that often this is attributed to prompt feedback received from the computer (Chai et al. 2014; Macaruso & Walker 2008; Scruggs 2008).

Multimedia technology is an aspect of computer-assisted teaching resource that has benefits across the learning styles of all types of learners as well as providing a different method of instruction (Campbell & Mechling 2009; Macaruso, Hook & McCabe 2006; Thompson 2005). Text-to-speech technology is one such multimedia technology that has the ability to convert electronic text to digitised speech. The technology has brought about the development of electronic literacy. Electronic literacy allows for all kinds of literary activities such as reading, writing and spelling that one can access through computers. Therefore, an important characteristic of multimedia is that it facilitates reading on every learner’s level (Thompson 2005).

This study assesses the influence of text-to-speech stories (also called talking books) on the reading ability of learners with ID. Talking books refers to audio-formatted recordings of books, magazines and other texts generally used to allow visually impaired learners to access such texts (American Foundation for the Blind 2016). The learners were put through a reading programme that involved listening to text-to-speech story on the computer. A pre-test was conducted prior to the intervention to assess the extent to which they could read at the time. A similar assessment was conducted after they had been exposed to the intervention (post-test). The learners and their teachers, who are all bilingual (English and Afrikaans), identified significant benefits from this intervention, especially within the context of special needs schooling in South Africa.

## Theory and literature

Literacy is an integral part of the curriculum. It is needless to say that success in other areas of learning is strongly linked to the extent to which learners can carry out the literacy acts of reading and writing (Erickson et al. 2009). In the case of learners with ID, literateness is regarded as a strong influence of how they are perceived because being literate enhances their face-to-face interaction with others (UNESCO 2006). In addition, increased levels of literacy among the intellectually disabled bring with them increased perceptions of competence from those without impairments.

While there are many theories with embedded concepts that are often tapped into in explaining learning and cognitive development, for this study we look specifically to Vygotsky (1978). Among other important contributions, his work explains learning among children who are chronologically of the same age but mentally on a different level. This difference between is what Vygotsky refers to as the zone of proximal development (ZPD). The ZPD takes into account the completed maturation processes as well as those that are taking shape. The concept implies that even in the absence of any intellectual impairment there is a distance between what learners know on their own and what they could potentially know if they receive the necessary assistance from the more capable other – be it technology or educators. In the case of this study, the assistance of the more capable other becomes even more crucial given the challenges imposed by the learners’ ID. Zone of proximal development therefore gives us insight into the joined supportive capabilities of technology and educators; and how this could influence the intellectually disabled learner’s learning to a desired level of functioning.

Zone of proximal development takes a new meaning when seeking to understand learning and development which does not occur as regularly theorised, such as in the case of those with ID. In this regard, we look specifically to Vygotskian theory of dysontogenesis (TD) which provides a framework for the analysis of enabling mechanisms for change with regard to the development of those with impairments. In this view, the strengthening and empowerment of skills as opposed to emphasis on weaknesses is the route to take for educators (Gindis 2003). To Vygotsky, mental functions (such as those involved in reading and learning how to in this instance) have their origin in interpersonal activity where ‘mental activity is mediated by culturally derived sign systems’ (Fernyhough 2008:227). This Vygotskian principle holds significant promise in the process of teaching learners with ID to read and acquire other scholastic tasks. In TD, disability is regarded as a social aberration (Lumadi 2013; Rodina 2006), and this view is held without necessarily discounting the biological properties of disability. The perspective clearly places the bulk of responsibility on the mechanisms educators employ for influencing change which could include how they incorporate different forms of mediating tools. Educators who understand and exercise the underlying principles of mediation are those who will seek culturally available means such as text-to-speech reading books in teaching reading. It is with this thought in mind that we explicate specific literacy needs of learners with intellectual impairments and implications of such needs on mediation.

### Specific needs and problems of the intellectually disabled learners

As explained earlier, ID imposes cognitive difficulties in reading, writing, spelling and the processing of numbers (American Psychiatric Association 2013b). Learners with ID typically have impairments in the following cognitive areas:

- Language, communication and auditory reception.
- Reasoning, idea production and cognitive speed.
- Memory and learning.
- Visual perception.
- Knowledge and achievement (Wehmeyer et al. 2004).

Edyburn (2004) has posited that one of the primary reasons for learners to be transferred to a special school is because of the difficulty they have with reading. More specifically, these affected learners struggle to meet the requirements of the reading and viewing learning outcome. Learners with ID struggle to memorise and rehearse text that they have read. They also struggle to organise text or instinctively elaborate on it to assist them to learn (Alfassi et al. 2009). Learners who have reading challenges also generally have difficulty in phonemic awareness and analysis, word identification, reading fluency and understanding of the text (Elder-Hinshaw et al. 2006). This brings us now to the literacy specific needs and challenges of such learners.

### Literacy-specific needs and challenges

Reading and writing constitute modalities through which language can be taught to young learners (Carstens 2013). Language can therefore be considered a tool for the conceptualisation and transfer of meaning and interpretation of texts (Silliman, Buttler & Wallach 2002; Wong, Graham, Hoskyn & Berman 2008). Reading according to Lessing and De Witt (2002) is a single aspect or learning outcome in literacy competence which can be described as the construction of meaning for which the learner must attain a necessary level of decoding proficiency. Children learn to read by progressing through a number of developmental processes. These are letter and word recognition, decoding, comprehension as well as how fluently the learner engages with the text (Long & Zimmerman 2009).

Word recognition refers to the ‘instant recall of words in which the reader resorts to no obvious mechanisms to recognize the word’ (Wong et al. 2008). When a learner is able to recognise words without hesitation, they have developed a state called automaticity that enables the reader’s brain to quickly and automatically process the words. In addition, learners who can instantly recall the words being read are capacitated with building mental representations of the message of the text that they can tap into when required to demonstrate their comprehension of a story (Allor et al. 2010).

Reading comprehension is the ability of the child to understand the text being read (Lea & Street 2006). Learners with reading difficulties generally experience poor comprehension because of their lack of being able to read and monitor their understanding of the information. The ability to decode words, poor vocabulary access and fluency in a language are contributory factors to a learner’s comprehension (Wong et al. 2008).

Reading fluency, being the last of the processes, relates to the speed and accuracy in the execution of the reading task (Chard Vaughn & Tyler 2002). The pace of a learner’s reading affects the way the learner retains the information and develops meaning from the text (Wong et al. 2008). Therefore, learners who read at a very slow pace struggle to retain the information being read, and consequently develop inadequate meaning from the text.

For a learner with ID, interventions that entail the teaching of language processes described above have been found effective in improving their reading abilities (Al Otaiba & Hosp 2004; UNESCO 2006). Given the literacy challenges, for example word identification, reading fluency and comprehension of the text, that the intellectually disabled learner has to contend with, what strategies can educators employ to ameliorate the literacy of such learners?

### Reading interventions

According to Edyburn (2004), when learners struggle to read, educators tend to resort to other methods of instruction. The problem is that a new method of instruction may not necessarily yield positive results. This is particularly true if the very problem of reading is part of the inherent nature of their disability. It is our view that, if one holds the position that all learners can learn, albeit at different levels, then any support mechanism or strategy would be worthwhile (DoE 2001; Gindis 2003). Among well-documented strategies is the use of assistive technology which is defined as ‘any item, piece of equipment or product system, […] used to increase, maintain, or improve functional capabilities of individuals with disabilities’ (Hobbs et al. 2009:153). The Foundation for Assistive Technology in the United Kingdom extends this definition to the ability to enhance ‘independence for disabled’. Brodwin, Star and Cardoso (2004:29) indicate that assistive technology does not only involve computers with all their components but involves an ‘integral process of assisting individuals with disabilities, […] to maximize their human potential’. Furthermore, computer assistive technology has due consideration for the learner and his or her individual traits, as well as his or her abilities and challenges. There are hosts of computer software on the market that can meet the specific needs of different groups of persons with a disability. In the section that follows, we highlight some of the technologies that have been used to aid learners with intellectual disabilities.

### Text-to-speech technology

Thompson (2005) refers to text-to-speech technology as a type of multimedia program that has the functionality of converting computer text to *digitised speech*. Zhao (2007:35) in turn indicates that speech technology refers to ‘technology that enables machines to receive and accept human oral language as input and respond with human or human-like oral language as output’. An important element of this technology is that it enables the learner to access software applications or content with immediacy in the speech feedback that could allow the learner to correct reading mistakes. Forgrave (2002) and Zhao (2007) are of the opinion that speech technology minimises the decoding problems that disabled learners sometimes have, which allows for better comprehension. Furthermore, it provides learners with repetitive visual and auditory cues that can help them to comprehend the text.

### Supportive e-text

E-text can be defined as the ‘text that has been altered to increase access and provide support to learners’ (Edwards 2008:36). Supportive electronic text aids learners with disabilities in dealing much better with text with the use and support of computer technology. One of the advantages of the computer software is the ability to change the way text is viewed and read, by modifying the font size and colour. The text can also be read aloud. Further to this, multiple images can be shown at any given time (Anderson-Inman & Horney 2007). These features are in contrast to printed text. The printed material in general does not afford the reader the opportunity to customise the text being read to them. Interestingly enough in instances of electronic text and printed text, the educator has to play a supportive role as well.

### Electronic books

E-books serve to replicate the printed paper-based storybooks into a digital format. In contrast to print paper-based books, e-books have additional multimedia effects to support the learner’s understanding of the text (Rhodes & Milby 2007). E-books have a number of intrinsic elements such as sound, animation and interactive activities. These elements can scaffold the learning of the learner, allowing him or her to eventually master the given task. In the process, the learner can be exposed to chunks of the reading task, for example to read one paragraph a few times then asking the learner to retell that part.

The interactive nature of e-books makes them particularly very attractive for young learners, and they tend to repeat activities which increase learning (Picton 2014). Learners with special educational needs, such as struggling readers, can therefore benefit from the additional text features of electronic books (Larson 2010). This particular feature could prove to be helpful for the participants of this study. The learners’ intellectual disabilities as has been indicated earlier range from mild to severe. This implies that the learners’ reading ability levels also vary. Multiple opportunities to expose the learners to text, which they have experienced on an auditory and a visual level, could aid their reading ability. ‘E-books and other text-to-speech readers boost students’ self-esteem while providing access to texts that were previously out of reach’ (Rhodes & Milby 2007:256).

Furthermore, children’s books that have been recreated into an electronic format allow the learner to track print and view a visual representation of the story. Electronic books help the learner to build their vocabulary, aid the understanding of the text while at the same time showing them how to read fluently (Horney & Anderson-Inman 1999; Rhodes & Milby 2007). However, some limitations do exist in the use of talking books. For example, e-books have limited use in the classroom during the course of the delivery of the literacy curriculum. Despite existing evidence from Chera (2002), some researchers are of the opinion that the true value of talking books having real educational potential has yet to be realised (Fox 2002; Littleton, Wood & Chera 2006). Research conducted by Chera (2002) has shown that talking books can promote phonological awareness in children during their initial reading experience of learning to read. Learners that have well-developed phonological awareness recognise on an auditory level that words can rhyme, start or end on the same letter and that letters can be manipulated to form new words. This unlocks future developmental skills that allow the learner to reflect and manipulate letters to create new words (Marthinussen 2011). Phonological awareness is regarded as a significant antecedent skill to the successful acquisition of reading (Adams 1990b; Blachman 2000; Goswami & Bryant 1990; Littleton, Wood & Chera 2006). Accordingly, the reader has to learn to master phonological skills that will enable him or her to break up the speech or the spoken word into phonological segments. It is clear from these studies that talking books have the potential to support reading development.

### Contextual enablers and constraints of technology in special needs education

Teaching children with intellectual impairments entails the use of appropriately identified technological aids. Text-to-speech technology is one such technologically intensive teaching aids in the context of special needs teaching and learning. This does not however mean that the decision to employ technology is taken blindly. We consider and accept the caution made by Roulstone (2016) that the use of technology, especially in special needs education, should be couched in the context of the learners. In the case of this study, the learners and the teachers are bilingual in English and Afrikaans. The use of text-to-speech technology is therefore suitable and easy to achieve because both English and Afrikaans are official languages in South Africa and are heavily used in many educational platforms. This consideration was important to negate the possibility of blindly adopting technological tools for the sake of technology, or to provide false hope of the potential of the tool to the learners (Breen 2015; Marchal-Crespo & Reinkensmeyer 2009).

## Research design and methodology

This paper reports on part of a larger qualitative action research that was conducted in 2013. The broader study sought to assess the impact of text-to-speech technology on the reading ability of intellectually impaired learners. We opted for a qualitative approach because our interest is in exploring how individual intellectually disabled learners responded to the technology, thus assessing the strengths of such technology as a mediating artefact.

**FIGURE 1:**
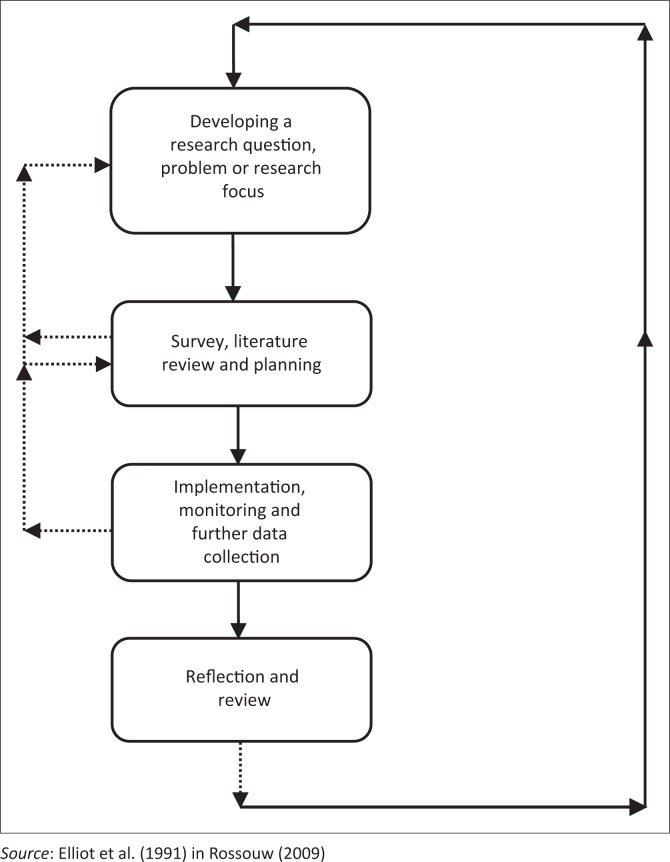
The action research process.

Action research is a ‘systematic study that combines action and reflection with the intention of improving practice’ (Cohen, Manion & Morrison 2007:297). In choosing action research intervention for this study, we consider issues such as the setting of the intervention, the participants involved and the theory that informs the analysis of the data. Bloomberg and Volpe (2008) explain that contextual information exposes the context within which the participants reside. The participants’ perceptions of the intervention is also important when analysing the data and the theory used to interpret the data should support the conclusions that are drawn and recommendations that are suggested (Bloomberg & Volpe 2008). For this, we adapted a four-step action research model from Rossouw (2009) (see Figure 1).

This model allowed us to base the implementation of our intervention on sound research, careful planning and alignment with our research questions. The strength of this model is that it enables us to use theory as a reflection tool when interpreting the data (Maxwell 2008). The underlying purpose of action was to involve educators in a continual interrogation of their practices on whether learners’ reading abilities were improving. In the process, the researchers and educators had the opportunity to possibly improve learning through informed, committed and intentional action (Beylefeld et al. 2007) in the form of repeated action of reading and recalling talking stories.

### Setting and participants

This action research took place at a special school within the Cape Metropole area that caters for learners with ID. The primary convenient sampling group comprised 35 learners across the five intermediate phase classes of the school where one of the researchers worked. To ensure that none of the learners was put at an advantageous or disadvantageous position in relation to one another, the following measures were undertaken: (1) all learners were exposed to the same reading activity, whether dependent (active educator support) or independent (less active educator support) and (2) no undue attention was granted to anyone beyond the level of support that would have been granted to all in the class during class activities. In this sense, the use of a control group was not an option so as to keep with ethical promises that all learners would be equally exposed to potential benefits of the intervention.

In addition to being in the intermediate phase (Grades 4–6), the learners met the following criteria:

- Prior to their participation in the study, the learners had been diagnosed as being intellectually disabled (the diagnosis of ID was carried out prior to placement at the special school) and were chronologically between 10 and 14 years old with an average mental age of between 4 and 7 years old.
- The learners were in the intermediate phase but were being exposed to the foundation phase (Grades R–3) curriculum because of their mental age.

The secondary sample group comprised the four educators of the learners. The reasoning behind the selection of this group was based on their daily direct involvement with the learners. The function of the educators was to assist the researchers to perform the pre- and post-tests as well as to engage the learners during the intervention sessions. The educators’ role in the study was therefore to provide qualitative accounts of learners’ progress or lack thereof.

The school selected for this study had the following contextual characteristics:

- well-resourced school (computer equipped with a computer lab and Internet),
- located within the Cape Town metropolis: access to excellent communication and technological facilities,
- bilingual classes (both learners and teachers use Afrikaans and English as a medium of teaching and learning).

### Procedure

#### Preliminary activities

Preliminary activities included securing permission to conduct the study from relevant authorities, applying for ethical clearance and piloting the study in classrooms similar to those that later served as the main study base. The content of the project was positioned within and aligned with the curriculum requirements of the school. The base curriculum was the national curriculum, and the project served as a form of differentiated teaching given the needs and strengths of learners at the school. Permission was sought from the educators to participate in the study as part of their day-to-day teaching activities and they were informed that participation was voluntary. One of the researchers’ roles was to set up all the study instruments and collection of data as he was an educator at the school. Parents’ consent was not sought because the project neither entailed anything outside the normal learning routines nor posed any form of danger or discomfort to learners.

The aim of the pilot was essentially to inform the main study. A pilot study is defined as a ‘small study conducted prior to a large piece of research to determine whether the methodology, sampling, instruments and analysis are adequate and appropriate’ (De Vos et al. 2002:211). The pilot study involved surveying the literature for guidance from previous research, consulting with experts in the field of education and technology and refining the data collection instrument.

The pre-tests and post-tests of the learners took place in the learners’ own classes. Pre- and post-testing was conducted with all five classes in the intermediate phase of the school. Each learner was given a short story from the Talking Story Series written by Margaret Koopedi (2007a–d) typed in a 28-point Century Gothic font. Learners were all given 1 min each to read the story consisting of 33–44 (Afrikaans language) and 34–35 (English language) words. The choice of which stories to read was determined by a learner’s home language (either English or Afrikaans). The learners’ reading behaviours including errors were noted and recorded for further analysis. Assessment of the learners’ reading was based on the following criteria: (1) words and sentences read, (2) total recalled words, (3) time utilised, (4) errors made and (5) other unfolding reading behaviours. The analysis of the learners reading across the pre- and post-test continuum followed the intervention sessions.

#### Interventions

**FIGURE 2:**
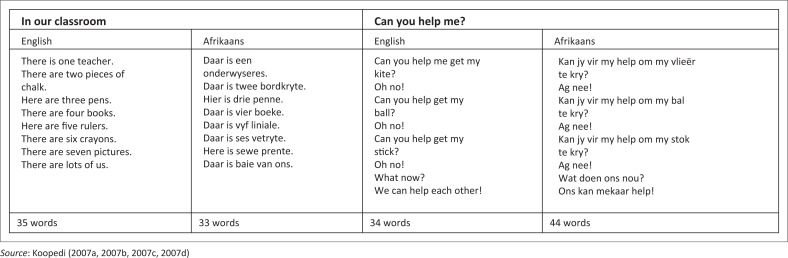
Intervention stories.

The interventions consisted of the learners being exposed to the same stories as had been used during the pre-tests. However, during the interventions the stories had text-to-speech elements included. Essentially, the learners made use of a text-to-speech program that was meant to stimulate them both visually and acoustically as opposed to printed information. In the process the learners could listen to electronic text on a computer screen whilst following the highlighted words at the same time. During the intervention the learners used headphones to listen to a story at least three times for 20 min. As the story was read by the computer, the individual words were highlighted. The stories were English and Afrikaans versions of ‘In our Classroom’ (*In ons klaskamer*) and ‘Can you help me?’ (*Kan jy vir my help*?) by Margaret Koopedi (Figure 2).

The learners were exposed to the words of the story on three levels. The first level was when the educator ‘read’ the story to them a few times and then following the story on the computer. The second level was when the individual learners got an opportunity to listen to, follow and read the story, whilst the rest of the group listened to their peers. The third level of exposure was when the educator explained and re-read the parts not properly read by the learners. This intervention was premised on research reported by Zhao (2007) and Thompson (2005). In both instances learners were exposed to books with text-to-speech capabilities during the intervention process. The focus was to improve the learners’ phonological (sound-symbol) ability as well as their recognition of words. The emphasis on word recognition is considered a crucial indicator to a learner’s understanding of text (Zhao 2007). Research reported by Littleton et al. (2006) had as a focus area the use of electronic text (text read from a computer monitor) as part of a reading programme. Demonstrations of how to operate the stories was carried out with the learners, after which they were allowed to use the books independently. An adult was always present during the course of the interventions. The learners were exposed to two computer sessions per book lasting about 15 min in total per week for a period of three months. They were post-tested after one week at the completion of a given intervention session. The duration of the project was three months as follows: April/May; May/June; and July/August.

### Educator interviews

Semi-structured interviews were conducted with the four educators helping with the tests’ administration and the intervention. The main purpose of the interviews was for the educators to share observations they had made about learners during the two processes. The interview questions were as follows:

- What in your view is the role of technology, especially computers, in the learning and teaching of mentally challenged learners?
- What, if any, have been the general achievements that your learners made when using computers as a learning tool?
- Have you observed any change in your learners’ reading ability whilst being part of the reading sessions? What have you learned from this change?
- How would you describe your learners’ reading competency (before and after the intervention) in relation to the following units:
  ■ the ability to code text
  ■ word recognition
  ■ visual discrimination of words
  ■ reading fluency
  ■ their understanding of the stories
- Do you have any last comments or observations?

### Ethical consideration

The study received ethical clearance through the CPUT Faculty of Education Ethics Committee (Approval Certificate EFEC 2-7/2009). Permission to conduct the study at Western Cape Public School was obtained from the Western Cape Education Department Research Directorate.

## Findings

The findings presented in this section are from reading performances of learners during pre- and post-tests and highlights from interviews conducted with educators of these learners.

### Reading performance

Two clearly defined groups emerged when performance was analysed. The first learner group are those who managed to read between 4 and 35 words, compared with the second group that had a very low to zero (0) reading pre-test score. From the outset it appeared that the strides made by learner group 1 (LG1) (high pre-test) was much higher than that of learner group 2 (LG2). That is, the learners who were able to identify more words during the pre-test were able to replicate and improve on that score during the post-test. In addition, time spent on reading (speed) by the LG1 was between 27 s and total given time of 60 s. The LG2 spent a full 60 s on the reading tasks. Beyond the question of how many words were read and how many errors were made, the next level of analysis focuses specifically on: (1) learners’ reading behaviours and (2) whether they made or did not make errors.

The behaviours and errors during reading were categorised along language processes and milestones expected of learners as they learn how to read. These are ‘letter and word recognition’, ‘decoding’, ‘comprehension’ as well as how fluently the learner engages with the text (Long & Zimmerman 2009:4). The categorisation was based on a constant comparison of all behaviours and errors. A feel or look alike criteria suggested by Maykut and Morehouse (1994) was used during the constant comparison to determine the categories. This process can be likened to the ‘first reading of, or first order imposition of meaning, on the data’ conducted in order to arrive at specific categories of unfolding descriptions of learner performance (Mosito 2005). The description of errors and reading behaviours follows.

### Description of reading behaviours and errors

- Omission: This refers to instances where a learner leaves out the words ‘pieces’, ‘of’, ‘chalk’ from the sentence ‘there are two pieces of chalk’.
- Substituting words in text with own words: A learner in this instance replaces the word ‘there’ with ‘here’ in the sentence ‘there are four books’. Another example was to read ‘pencils’ instead of ‘pictures’ in the sentence ‘here are seven pictures’.
- Refusal to read: The learner refuses to read any part of the story and keeps quiet for the remainder of the session. No reading took place.
- Identification of letters as opposed to whole words: It means the learner identifies certain or all the letters in a given word instead of reading the whole word, for example, ‘o’, ‘e’, in the word ‘onderwyser’ (educator).
- Self-corrects misread words: An example of this is where a learner (e.g. Rhona and Bradley) reads ‘onderwyser’ (male educator) and corrects the word by reading ‘onderwyseres’ (female educator). This implies that learner has realised that he or she has omitted a segment of a word and corrected it without prompting.
- Reversals: A learner tends to read letters or words in reverse (Ekwall 1981), for example ‘d’ for ‘b’ in the word ‘daar’ (there) read as ‘baar’ in the sentence ‘daar is een onderwyseres’ (there is one educator).
- Additions or insertions: The learner, for example, adds a word in the sentence ‘daar is twee bordkryte’ (there are two pieces of chalk) reads as ‘daar is net twee bordkryte’ (there are *just* two chalks). Often these additions do not distort the meaning of the sentence.

In line with the above descriptions, Table 1 depicts the performance of LG1.

**TABLE 1:** Learner group 1 (LG1) categorisation of reading behaviours.

Category	Errors and behaviours	Pre-test incidences	Post-test incidences
Word and letter recognition	1,2,3,4,5 and 6	201	75
Comprehension	7 and retell	101	211
Fluency	Words read Sentences read Seconds on task Errors made	Words: 146 Sentences: 44 Time: 560 Errors: 192	Words: 247 Sentences: 65 Time: 481 Errors: 91

*Source*: Authors’ own work

It is clear from Table 1 that the most dominant feature of LG1 reading behaviour was problems with word and letter recognition. On the positive side, there was a notable decrease in word and letter recognition problems during the post-test. In addition, during the post-test some of the learners (3) indicated comprehension of the story by adding correctly to the context words(s) which were not in the story they were asked to retell.

The positive behaviours noted in Table 1 are corroborated by educators’ observations as they responded to questions on (1) general achievements and (2) specific reading behaviours with regard to reading processes such as word recognition, comprehension and fluency. On the first question, there was consensus among the four educators that computer technology seemed to facilitate independence and discipline. One of the participating educators commented,

‘I pick up a tremendous enthusiasm in the learners … they insist they want to read every day … they longed for what they have learnt … means a lot for them … I must just make it smaller … they must get the exercise everyday… when we go to the computer lab … they want to go straight to the stories … good thing [*Text translated from Afrikaans*].’ (Educator 4, female, LG1)

In Table 2, findings from the reading performance of the remaining 26 learners are presented.

**TABLE 2:** Learner group 2 (LG2) categorisation of reading behaviours.

Category	Errors and behaviours	Pre-test Incidences	Post-test incidences
Word and letter recognition	1,2,3,4,5 and 6	734	628
Comprehension	7 and retell	44	213
Fluency	Words read Sentences read Seconds on task Errors made	Words: 6 Sentences: 2 Time: 1560 Errors: 765	Words: 104 Sentences: 37 Time: 1560 Errors: 664

*Source*: Authors’ own work

Similarly to the LG1 in Table 1, LG2 also demonstrated high instances of word and letter recognition difficulties. The incidence of these difficulties improved slightly during the post-test. Five of the learners demonstrated comprehension of the story through their ability to add a correct word to the initial story. One of the observations made by the researcher-educator was that the errors that were made by LG2 were in many ways far removed from the real word or were just nonsensical. These types of errors were based on the fact that the learner could not decode the requested word. This observation explains the slight decrease in errors noted between the pre- and post-tests.

As the study was not about a performance of a particular group against the other (LG1 vs LG2), in Table 3 all the behaviours per category from both groups was added in order to arrive at the overall picture of all the learners. These totals inform the discussion that follows.

**TABLE 3:** Overall categories of reading behaviour.

Category	Errors and behaviours	Pre-test	Post-test
Word and letter recognition	1,2,3,4,5 and 6	935	703
Comprehension	7 and retell	145	424
Fluency	High total word count; High total sentence count; Fewer errors made; High retell word count; Less time used.		LG1 improved on all criteria LG2: some improved slightly in a, b, c and d criteria but did not improve on time (e)

*Source*: Authors’ own work

LG1, Learner group 1; LG2, Learner group 2.

It is clear from the above overall depiction of learner performance between the pre- and post-tests that there was a positive upward movement in learners’ comprehension of the story. The positive trend is indicated by a high level of meaningful insertions of words which did not feature in the actual story words and the learners’ ability to retell the stories. Educators’ observations of learner performance were in line with the trend. For example, Educator 3 disclosed

‘… Word recognition, … sight reading … and now I see with some of them one could move beyond that; visual discrimination of words, … showing me word for word, … using that method, … found that it works well for me.’ (Educator 3, male, LG1 & 2)

Another important observation made by the educators was that the text-to-speech artefact had been the most exciting part of the interventions. Educator 1 explained that stories told and seen through the computer seemed to generate an interest level she had not seen in learners before. In her own words, ‘a child views a story on the computer, hearing this voice coming from the computer … makes learning interesting and fun’ (Educator 1, female, LG1 & 2).

With the picture portrayed by the findings in Table 3, we are now at an informed position to address more specifically the question of the nature of change in reading abilities learners with ID in this study have undergone following teaching that was mediated with text-to-speech books.

## Discussion

The gains made by learners might appear insignificant if we were concerned with a learner population without ID. For this group of learners gains like increased enthusiasm and motivation, observable increase in number of words read and sophistication in comprehension such as insertions of their own meaningful words in retelling the story could all attest to the joined mediational impact of educators and listening to and seeing story words on the computer. As reading ability consists of several processes ranging from word recognition, comprehension and fluency, we examine more specifically the nature of processes that emerged following the intervention.

### Word recognition

The biggest reading difficulty experienced by learners in this study pertained to word recognition. Allor et al. (2010:4) state that ‘good readers effortlessly recognize words and build mental representations of the message of the text’. This implies that learners who struggle with word recognition will be robbed of the tools for comprehending and storing messages for later recall. This finding leads to a conclusion that word recognition is a major challenge for intellectually disabled learners. While there appears to have been improvement among one group of learners in this instance, the duration of the intervention might not have allowed for high levels of achievement across all participating learners.

### Comprehension of text

Where reading comprehension is concerned, the performance of learners in this study confirms what previous research has revealed. Learners with word recognition difficulties have lower grasp or comprehension of the story which is often exacerbated by poor decoding required for word recognition (Wong et al. 2008).

### Reading fluency

A number of correct words and sentences read and time spent on reading are indicative of reading fluency which is defined as the speed and accuracy in the execution of the reading task (Chard, Vaughn & Tyler, 2002). Another finding in this study is the extent to which the participating learners were challenged in this process and improved or did not between the pre- and post-tests. The analysis resulted in two distinct groups of learners in this regard (LG1 and LG2). The former group’s performance marked them as the stronger lot as they utilised far less of the provided reading time and some managed to read all the 35 words. Most of the participants in LG2 utilised all the given time of 60 seconds but failed to read a single word correctly.

### Limitations of the study and future research

It is on the question of the emerging reading processes that one limitation of the study reveals itself. Initial in-depth analysis of data should have occurred after the pre-test in order to isolate those areas that the intervention could have addressed. This limitation is a crucial finding by itself which future research could address. The second limitation relates to the absence of a control group. It is possible that what we have interpreted as reading gains could have resulted from the use of any teaching artefact besides the text-to-speech stories. Performance of a control group which was not exposed to the intervention could have led to clearly established conclusions. Lastly, that the analysis isolated two groups and this was not followed up, for example in terms of probing from the educators concerned if they did anything differently which in their opinion could have resulted in higher gains for LG1, was a lost opportunity for revealing the specific variables that could have added to this marked difference between the two groups.

## Conclusion

The study set out to assess the influence of computer technology on reading abilities of learners with intellectual impairment. Two crucial implications can be drawn from the foregoing discussion: Despite initial intents, no claims of this influence can be made given the duration of the intervention and the non-exclusion of other influences within the study context which could have influenced the reading behaviours noted in the foregoing section. All the same, important learnings have unfolded from the study.

Firstly, the study clearly points towards the potential of computer-assisted learning in isolating reading processes that interventions based on testing could address. An example is increased word recognition which ultimately leads to reading fluency. The second crucial learning is the role of computer technology in enhancing enthusiasm and motivation to learn which in a sense supports findings from similar studies (Scruggs 2008). When all the factors are put together, it is clear that many positive attributes associated with teaching and assessing reading through talking stories position this form of technology as a necessary tool in the education of learners with ID. This study has successfully applied the social-constructivist notion of ‘remediation and compensation for abnormal development’ (Gindis 2003; Yankun 2006) such that the learners’ reading competencies as well as their attitudes towards reading have to some extent improved. The results support Vygotsky’s call for education planning and design based on inclusion, and positive differentiation (Karpov 2005).
